# Utility of a dual immunostain cocktail comprising of p53 and CK20 to aid in the diagnosis of non-neoplastic and neoplastic bladder biopsies

**DOI:** 10.1186/1746-1596-4-35

**Published:** 2009-10-14

**Authors:** Isil Z Yildiz, Rosemary Recavarren, Henry B Armah, Sheldon Bastacky, Rajiv Dhir, Anil V Parwani

**Affiliations:** 1 Department of Pathology, Shadyside Hospital, University of Pittsburgh Medical Center, Pittsburgh, Pennsylvania, USA; 2 Department of Pathology, Presbyterian University Hospital, University of Pittsburgh Medical Center, Pittsburgh, Pennsylvania, USA

## Abstract

**Background:**

Distinction between non-neoplastic and neoplastic bladder lesions is therapeutically and prognostically important. Our objective is to describe the use of double immunohistochemistry (DIHC) for p53+CK20 as a tool for diagnosing neoplasia in bladder biopsies.

**Methods:**

p53+CK20 DIHC were examined in 38 reactive atypia, 10 dysplasia, 9 carcinoma in situ (CIS) and 7 invasive carcinoma (IC) cases. CK20 was evaluated according to distribution extent and degree of intensity whereas percentage of positive cells together with staining intensity was taken into account in the evaluation of p53.

**Results:**

92% of reactive cases were either CK20(-) or (+) only in the upper 1/3 urothelium. In dysplastic cases CK20 staining distribution was as follows: 60% in 2/3 of the urothelium, 30% full thickness, 10% in the upper 1/3 urothelium. Among CIS cases, 89% had full thickness CK20 positivity, of which 62% were p53(+). 71% of IC cases exhibited strong and full thickness dual staining.

**Conclusion:**

This is the first study in the literature to use DIHC of p53+CK20 in distinction of non-neoplastic and neoplastic bladder lesions. Dual staining by p53+CK20 cocktail allows for histologic correlation and diminishes the risk of losing the area of interest in limited biopsy specimens.

## Background

Urothelial carcinoma is the 7^th ^most common cancer worldwide with 260,000 new cases in men and 76,000 cases in women per year [1]. Furthermore, it is the 4^th ^leading cause of cancer in American men with 14,100 deaths per year in the US [2].

2004 World Health Organization/International Society of Urological Pathology (WHO/ISUP) consensus committee classifies flat urothelial lesions with atypia as reactive urothelial atypia (RUA), atypia of unknown significance (AUS), urothelial dysplasia (UD) and carcinoma in situ (CIS) [1,3]. UD is defined by "appreciable loss of polarity with nuclear rounding and crowding and cytologic atypia that is not severe enough to merit a diagnosis of CIS." whereas AUS is a descriptive category for cases in which dysplasia cannot be ruled out for sure, because of a degree of atypia that is discordant with the extent of inflammation [1]. Both UD and CIS are precursor lesions of invasive carcinoma and their presence is associated with a high risk of progression and recurrence [3-5]. Although the morphologic criteria is very useful, CIS diagnosis may be challenging in cases where the reactive/therapy atypia is in the differential diagnosis. Generally, in reactive atypia, polarity is maintained and accompanying inflammation or history of reactive conditions (calculi, trauma, instrumentation or infection) may be present and cells lack irregular chromatin distribution and pleomorphism. The presence of large, irregular, hyperchromatic nuclei, large nucleoli, and frequent mitoses including atypical ones in the midurothelium to upper urothelium raise the possibility of CIS [1]. However, some patterns of CIS lack pleomorphism and may mimic reactive atypia [6]. CIS cases with scattered atypical cells and pagetoid spread may be underdiagnosed because of the absence of full-thickness atypia and may be overlooked as non-neoplastic lesions [7].

Persistent UD or CIS in a background of therapy is considered as therapy failure and may lead to radical cystectomy. Therefore differentiating UD and CIS from RUA in the background of inflammatory/post-therapy changes is critical because of appearent therapeutic and prognostic implications. Other reasons such as small specimen size and interobserver differences may also contribute to difficulties in making the correct diagnosis [7]. Morphology alone may not be sufficient in the differentiation. Hence, in diagnostically difficult cases of AUS, specific markers of UD and CIS to enhance morphology would be of great utility to pathologists in distinguishing RUA from UD and CIS.

In the search for reliable markers; p53 and cytokeratin 20 (CK20) are emerging as useful indicators of neoplastic change and prognosis in urothelial proliferations as the reports in favor of them accumulate [8-12].

## Methods

64 bladder biopsies, consisting of 38 benign/reactive, 10 dysplasia, 9 CIS, and 7 invasive carcinoma (IC) (6 papillary, 1 flat) cases, were retrieved from our surgical pathology files. The samples were fixed in 10% buffered formalin solution and embedded in paraffin blocks. The biopsies were diagnosed according to the 2004 WHO classification of tumors of the urinary system [1].

Sections (4 μm) from each case were obtained. Deparaffinization, rehydration, and antigen unmasking were achieved by boiling sections in a commercially available steamer. After quenching endogenous peroxidase, slides were incubated with a cocktail of p53+CK20 composed of 10 ul of anti-p53 polyclonal antibody (1:500; DAKO, Carpinteria, CA), 50 ul of anti-CK20 monoclonal antibody (1:100; DAKO, Carpinteria, CA) and 4,940 ul of Van Gogh Yellow diluent. The detection system used was a double stain polymer detection kit.

H&E and double immunohistochemistry (DIHC) of p53+CK20 were examined for each case. Nuclear staining for p53 and cytoplasmic staining for CK20 were interpreted as positive. p53 was evaluated according to the ratio derived from the number of p53-positive cells and staining intensity and scored as: 0 (no staining), 1+ (<15% cells, weak), 2+ (15-50% cells, moderate), 3+ (>50% cells, strong) by semi-quantitative reassortment. CK20 cytoplasmic stain was also semiquantitatively evaluated: 0 (no staining), 1+ (patchy, weak), 2+ (<50% cells, moderate), 3+ (>50% cells, strong). CK20(+) cells were additionally described by location: Upper 1/3 urothelium including umbrella cells, 2/3 urothelium sparing basal layer and full-thickness staining including the basal layer.

## Results

**Figure 1:**
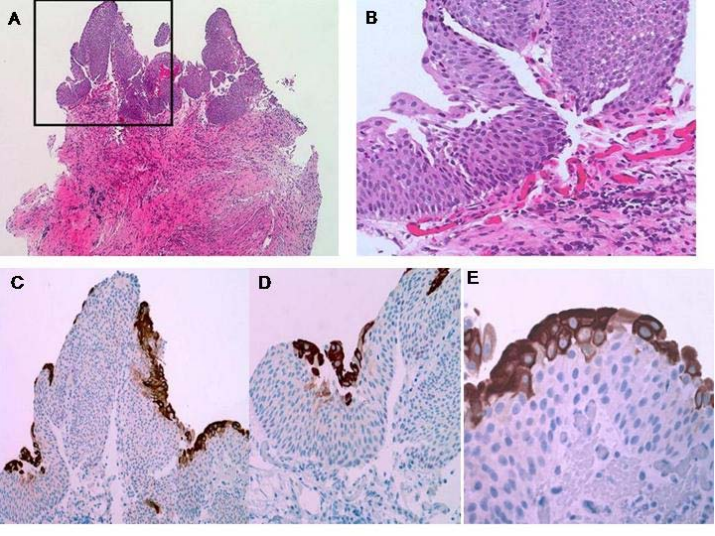
**Reactive urothelium (A and B) (hematoxylin eosin, original magnifications × 40 (A), × 200 (B))**. Cytoplasmic immunoreactivity in reactive urothelium to CK20 in 1/3 urothelium including umbrella cells (original magnifications × 40 (C), × 200 (D), × 400 (E)).

35 out of 38 (92%) benign/reactive cases were either CK20(-) or showed CK20 positivity only in the upper 1/3 urothelium including umbrella cells and the majority, 21 out of 35 (60%), of these cases were p53(-). The remaining 3 out 38 (8%) cases showed variable CK20 staining wheras 14 out of 35 (40%) showed weak (1+) p53 positivity (Figure 1).

**Figure 2:**
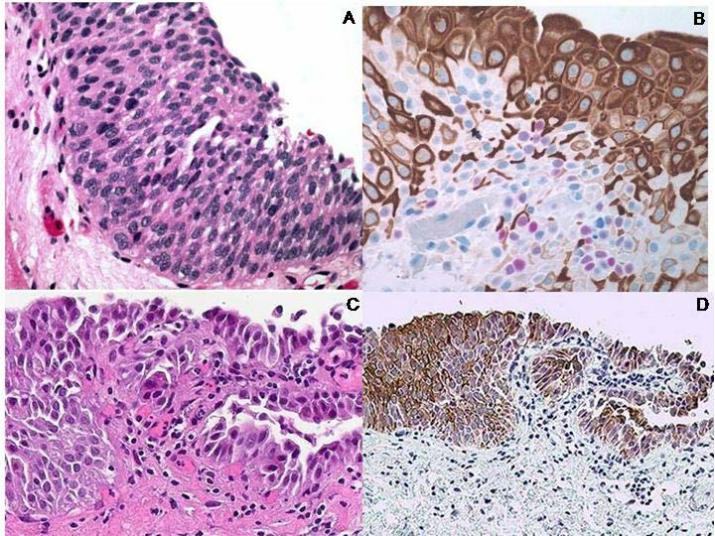
**Dysplastic urothelium (A and C) (hematoxylin eosin, original magnifications × 200)**. Cytoplasmic immunoreactivity in dysplastic urothelium to CK20 in 2/3 of the urothelium sparing the basal layer and nuclear 2+ immunoreactivity to p53 (original magnification × 200 (B)). Cytoplasmic full-thickness immunoreactivity in dysplastic urothelium to CK20 (original magnification × 100 (D)).

Among dysplastic cases, 6 out of 10 cases (60%) exhibited CK20 positivity in 2/3 of the urothelium sparing the basal layer while 3 cases (30%) showed full-thickness CK20 positivity, only 1 of which was accompanied by 3+ p53 positivity and 1 out 10 dysplastic (10%) cases was CK20(+) only in 1/3 superficial urothelium (Figure 2).

**Figure 3:**
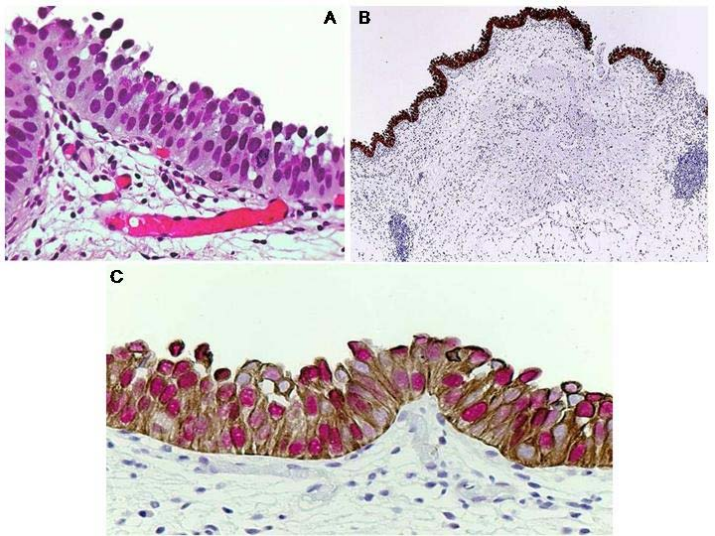
**CIS (carcinoma in situ) in urothelium (A) (hematoxylin eosin, original magnification × 400)**. Cytoplasmic full-thickness immunoreactivity to CK20 and nuclear 3+ immunoreactivity to p53 (B and C) (original magnification × 40 (B), × 400 (C)).

8 of 9 (89%) CIS cases showed focal or diffuse full-thickness CK20 positivity and 5 out of those 8 (62%) were 3+ p53(+). 1 of the CIS cases (11%) showed CK20 positivity only in the upper 1/3 urothelium accompanied by strong 3+ p53 positivity involving the basal layer (Figure 3).

**Figure 4:**
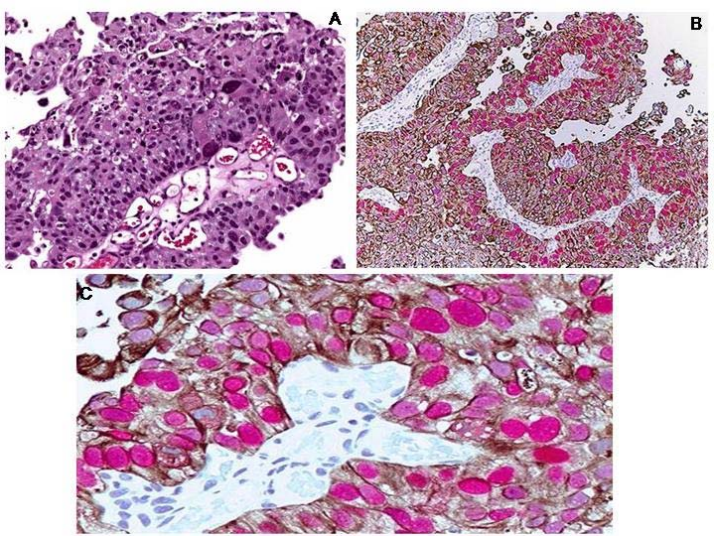
**Invasive carcinoma (A) (hematoxylin eosin, original magnification × 200)**. Cytoplasmic full-thickness immunoreactivity to CK20 and nuclear 3+ immunoreactivity to p53 (B and C) (original magnification × 100 (B), × 400 (C)).

Among invasive carcinoma cases, 5 of 7 (71%) were full-thickness CK20 positive, 2 of those cases (40%) showed 3+ p53 positivity whereas other 2 cases (40%) showed 2+ p53 positivity. The remaining 2 out of 7 (29%) cases showed CK20 positivity other than full thickness whereas 3 out of 7 (43%) cases were p53(-) or showed weak p53 positivity. In dually positive cases, CK20 and p53 occupied the full-thickness urothelium, including the basal layer (Figure 4).

**Figure 5:**
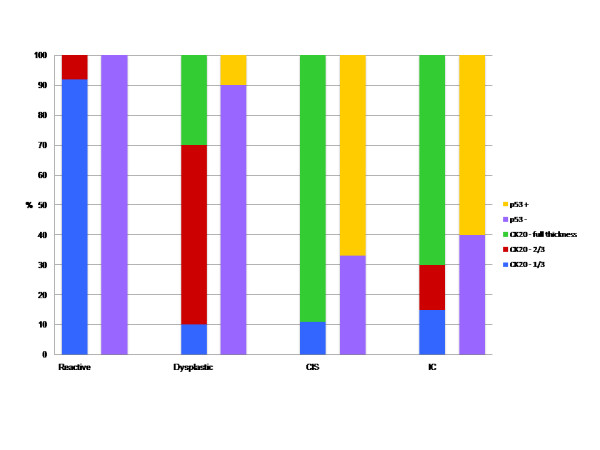
**The distribution of location of CK20 staining in the urothelium and presence of p53 staining (0 and 1+ staining = p53 -, 2+ and 3+ staining = p53 +) according to diagnosis (CIS: carcinoma in situ and IC:invasive carcinoma) groups**.

The distribution of location of CK20 and presence of p53 staining according to diagnosis groups are given on Figure 5.

## Discussion

The problem of distinction between reactive and dysplastic atypical changes in the urothelium has not been resolved, even after 2004 WHO/ISUP classifications [1,3]. Much inter and intraobserver disagreement constitute a dilemma due to lack of definite morphological criteria to diagnose CIS, dysplasia and reactive atypia.

CIS is a high-grade intraurothelial neoplasm. Patients with CIS are at significant risk for the development of invasive urothelial carcinoma, cancer recurrence, progression, and even death from bladder cancer [3-5,13,14]. Therefore the recognition of CIS in bladder biopsy specimens is of great importance because it not only has important prognostic implications but may also alter subsequent therapy. The histopathology of urothelial CIS may overlap with RUA resulting in diagnostic difficulty when interpreting bladder biopsies [9]. The difficulty and importance of distinguishing CIS from benign atypias should not be underrated as malpractice cases involving the failure to recognize CIS are growing in number [15]. Dysplastic intraepithelial lesions are another problematic area that requires distinction from CIS and which pathologists frequently feel uncomfortable evaluating on the basis of histological criteria alone.

As the distinction is therapeutically and prognostically critical, objective ancillary markers to help in the histologic differentiation between CIS and dysplasia and reactive atypia are necessary. Importance of special and immunohistochemical stains for correct diagnosis and accurate subclassification of controversial cases in other lesions of urinary tract are also emphasized recently by Jin et al [16] and Pradhan et al [17]. Previous studies have demonstrated the diagnostic utility of separate p53 and CK20 immunohistochemistry (IHC) in assessing neoplasia in bladder biopsies [11]. These markers are known to be easy to manage and interpret. Distinctive immunoreactivity patterns with antibodies against p53 and CK20 were identified for reactive atypia and CIS [9]. Abnormal CK20 expression in urothelial cells accompanied by overexpression of p53 are considered indicators of dysplastic change in urothelial mucosa and immunostaining with p53 and CK20 may help accurately diagnose CIS [11,18].

To our knowledge, no previous study in the literature has used DIHC of p53+CK20 in distinction of non-neoplastic and neoplastic bladder lesions. In this study, we investigated the utility and advantages of p53+CK20 DIHC as a tool for detecting synchronous expression of both markers in bladder biopsies and for objectively distinguishing the cases with CIS and dysplastic urothelial changes from reactive nonneoplastic atypia.

The nuclear overexpression of p53 by immunohistochemistry has been shown to be representative of p53 tumor suppressor gene mutation which is a common event in neoplastic urothelium [19-26]. p53 is considered to be nonexpressed in the nonneoplastic urothelium and overexpression of the p53 gene product has been reported as a marker of progression in urothelial carcinoma [24,25,27-29]. Mutations of the p53 gene and immunohistochemical positivity for the p53 protein have been found in 40% to 60% of urothelial carcinomas in studies of Olumi, Sidransky and Wright et al. [21,28,30]. In nonneoplastic and reactive urothelium, expression of p53 has been described as varying from negative to weak and patchy, predominantly in basal and parabasal intermediate cells [9,11,31]. In parallel to these results, 60% (21/35) of the benign/reactive group cases of our study were found to be p53(-). In contrast, p53 was considered positive by Mallofre in 80% of the CIS cases, with 70% of those cases showing positivity in 50% of the cells [11]. Similarly, McKenney found that p53 was positive in 57% of the CIS cases, with all of the cases exhibiting positivity in more than 50% of malignant cells [9]. In our study, 10% (1/10) of the dysplasia, 67% (6/9) of CIS and 29% (2/7) of invasive carcinoma cases showed strong diffuse p53 positivity whereas another 29% (2/7) of invasive carcinoma cases exhibited moderate p53 positivity.

If found in the cytoplasm of cells different than superficial umbrella cells and occasional intermediate cells, CK20 is considered abnormally expressed as a marker of abnormal urothelial differentiation [31,32]. Urothelial de-differentiation, as with neoplastic change, is accompanied by expression of CK20 in all cell layers as shown by Harnden et al. [8,32]. This change in the extent of expression makes CK20 a useful and reliable marker of the neoplastic change of urothelial cells [8,32-36]. Immunostaining for CK20 is therefore an important addition to morphology in the diagnosis of neoplasia, especially, in the differentiation from reactive states where diagnostic difficulties are greatest [8,37]. Cases of atypia and dysplasia that display abnormal CK20 staining should raise the possibility of CIS and be followed up appropriately. In the studies of Mckenney, Mallofre and Kunju et al it has been reported that in nonneoplastic epithelium as well as the reactive urothelium CK20 showed patchy cytoplasmic immunoreactivity in only the superficial umbrella cell layer [9,11,37]. In favor of those studies, in our study, we found that 92% (35/38) of benign/reactive cases were either CK20(-) or showed CK20 positivity only in the upper 1/3 urothelium. While confined to superficial umbrella cells in normal urothelium, CK20 has been reported to show diffuse full thickness staining in 72-89% of cases of CIS and has also been reported to be expressed in 22-58% of invasive urothelial carcinomas [7,9,11,37-39]. However, CK20 immunoexpression was found to be nondiscriminatory in 11-28% of CIS cases due to lack of CK20 expression [7,9,11,37]. In this study, abnormal expression of CK 20 was found in 90% (9/10) of dysplasia, 89% (8/9) of CIS and 71% (5/7) of IC cases whereas the rest of the cases lacked abnormal CK20 expression.

## Conclusion

According to our results, we can conclude that our proposed immunohistochemical panel composed of p53 and CK20 for studying dysplastic urothelial changes is adequate and useful for confirming the presence of dysplastic changes in the urothelium and can be of aid in better defining the histological criteria of urothelial dysplasia.

Furthermore, our studies have demonstrated that p53+CK20 cocktail (if commercially available) may be a useful diagnostic marker in the assessment of bladder biopsies. Two markers, in our opinion, could be used in routine practice, together with careful clinical and morphologic correlation. p53+CK20 DIHC can be considered as a useful innovation in differentiating non-neoplastic vs. neoplastic lesions in bladder biopsies enhancing the credibility of the diagnosis and reducing the number of inconclusive pathology reports of atypia of unknown significance consequently minimizing the need for rebiopsy as the dual staining not only allows for histologic correlation but also diminishes the risk of losing the area of interest in limited biopsy specimens in recut sections.

## Abbreviations

DIHC: Double immunohistochemistry; CIS: Carcinoma in situ; IC: invasive carcinoma; WHO/ISUP: World health organization/international society of urological pathology; RUA: Reactive urothelial atypia; AUS: Atypia of unknown significance; UD: Urothelial dysplasia; IHC: Immunohistochemistry.

## Competing interests

The authors declare that they have no competing interests.

## Authors' contributions

IZY collected the background references, wrote the discussion of the results, created the sequence of alignment and drafted the manuscript. RR obtained the diagnostic material from the archive of the pathology department, carried out the immunohistochemical tests and pathological examination, took the photomicrographs and displayed the results of the study. HBA contributed to the preparation of the manuscript. SB, RD and AVP participated in the design and coordination of the study and gave and reviewed the final histopathological diagnosis. AVP revised the manuscript. All authors read and approved the final manuscript.
